# IGHG1 upregulation promoted gastric cancer malignancy via AKT/GSK-3β/β-Catenin pathway

**DOI:** 10.1186/s12935-021-02098-1

**Published:** 2021-07-27

**Authors:** Xinyu Li, Wen Chen, Chunkang Yang, Yisen Huang, Jing Jia, Rongyu Xu, Shen Guan, Ruijun Ma, Haitao Yang, Lifeng Xie

**Affiliations:** 1 Department of Gastrointestinal Surgery, Quanzhou First Hospital Affiliated to Fujian Medical University, Quanzhou City, 362002 Fujian Province China; 2 Department of Traditional Chinese Medicine Oncology, The Second Affiliated Hospital of Fujian Medical University, Quanzhou City, 362000 Fujian Province China; 3 Department of Gastrointestinal Surgery, Fujian Cancer Hospital and Fujian Medical University Cancer Hospital, 420 Fuma Road, Jin’an District, Fuzhou City, 350005 Fujian Province China; 4 Department of Thoracic Surgery, Quanzhou First Hospital Affiliated to Fujian Medical University, Quanzhou City, 362002 Fujian Province China; 5 Department of General Surgery, Tongxin County People’s Hospital, Ningxia Hui Autonomous Region, Wuzhong City, 751300 Tongxin County China; 6 Department of General Surgery, Wuzhong People’s Hospital, Ningxia Hui Autonomous Region, Wuzhong City, 751000 China

**Keywords:** Gastric cancer, IGHG1, β-Catenin

## Abstract

**Background:**

Despite current advances in gastric cancer treatment, disease metastasis and chemo-resistance remain as major hurdles against better overall prognosis. Previous studies indicated that IGHG1 as well as -Catenin serve as important regulators of tumor cellular malignancy. Therefore, understanding detailed molecular mechanism and identifying druggable target will be of great potentials in future therapeutic development.

**Methods:**

Surgical tissues and gastric cancer cell lines were retrieved to evaluate IGHG1 expression for patients with or without lymph node/distal organ metastasis. Functional assays including CCK8 assay, Edu assay, sphere formation assay and transwell assay, wound healing assay, etc. were subsequently performed to evaluate the impact of IGHG1/-catenin axis on tumor cell proliferation, migration and chemo-resistance.

**Results:**

Gastric cancer tissues and tumor cell lines demonstrated significantly higher level of IGHG1. Functional study further demonstrated that IGHG1 promoted proliferative and migration as well as chemo-resistance of gastric cancer tumor cells. Further experiments indicated that IGHG1 activated AKT/GSK-3/-Catenin axis, which played crucial role in regulation of proliferative and chemo-resistance of gastric cancer cells.

**Conclusion:**

This study provided novel evidences that IGHG1 acted as oncogene by promotion of gastric cancer cellular proliferation, migration and chemo-resistance. Our research further suggested that IGHG1/AKT/GSK-3β/β-Catenin axis acted as novel pathway which regulated gastric cancer cellular malignant behavior. Our research might inspire future therapy development to promote overall prognosis of gastric cancer patients.

**Supplementary Information:**

The online version contains supplementary material available at 10.1186/s12935-021-02098-1.

## Background

Globally speaking, gastric cancer stands as one of the most common cause of cancer mortality. According to the latest statistical data, gastric cancer remains as the fifth most common cancer. It has been clarified that east Asia, eastern Europe and south America are geographic hotspots for gastric cancer occurrence [1]. Several aspects including H. Pylori infection, age, salt intake, low vegetable intake are confirmed as risk factors for gastric cancer pathogenesis [2]. Up to date, the main therapeutic strategy for early gastric cancer is endoscopically surgical resection. And neoadjuvant chemotherapy significantly promoted gastric cancer patients with advanced stages. However, as a molecularly heterogeneous type of cancer, therapeutic effects among different patients varies depending on disease complex genetic background. Therefore, further understanding the underlying mechanism of metastasis and chemo-resistance is of vital importance in enhancement of disease prognosis and therapeutic effects.

As a fundamental component of human adaptive immunity, immunoglobulin G (IgG) consists around 80 % of total immunoglobins (IgGs) [3, 4]. Despite the fact that only B cells and plasma cells are known to be capable of IgG production, accumulating reports demonstrated that several kinds of malignant cells could generate IgGs, such as tumor cells from esophageal, breast, liver or prostate, etc. [5–7]. Current studies indicated that IgGs exhibit promotive effects on tumor expansion and invasion in multiple malignancies. It is still not known the role of IgGs in gastric cancer.

β-Catenin acts as a central regulator in multiple physiological processes, and its dysregulation has been implicated in different types of cancers [8, 9]. β-Catenin regulates transcriptional activities of various genes and cellular adhesion. Wnt protein regulates the activity of β-Catenin through canonical pathways via multiple signal transducers including glycogen synthase kinase 3 beta (GSK3β), adenomatous polyposis coli (APC), Axin and T cell factor (TCF)/lymphoid enhancement factors (LEF) [10–12].

In this study, we aimed to explore the role of IgG1 heavy chain constant region (IGHG1) in the malignant biological behaviors of gastric cancer cell. And we also further delineated the regulatory role of IGHG1 on Wnt/β-Catenin pathway, in order to identify potential target for future gastric cancer treatment.

## Methods

### Patient recruitment and sample collection

This study was approved by the Ethics Committee of Quanzhou First Hospital Affiliated to Fujian Medical University and informed consent was obtained from the patients. Patients who were diagnosed with gastric cancer in our clinical center from Feb, 2019 to Aug, 2020 were retrospectively enrolled in this study. No prior surgical treatment, radiotherapy or chemo-therapy were conducted. Clinical samples of gastric cancer tissues and adjacent normal tissues were gathered post-surgical treatment. Samples were stored in liquid nitrogen for subsequent experiments.

### RNA extraction and qRT-PCR experiments

Total RNA samples from cell line samples and clinical tissue were extracted by RNAiso Plus agent (TaKaRa, Dalian, China) according to the standardized protocol. The level of RNA was quantified and reverse transcription polymerase chain reaction was performed to generate cDNAs. The levels of mRNA expression were quantified by real-time PCR qRT-PCR reaction with condition setting as 94 °C for 30 s, 55 °C for 30 s, 72 °C for 90 s, with total cycles of 40. Primers used in this study were listed in detail (Additional file 1: Table S1).

### Cell line culturing

Gastric cancer cell lines (MKN45, HGC27, MGC-803, BGC-823, AGS, MKN28) and human normal gastric epithelial cell (GES-1) were purchased from American Type Culture Collection (ATCC; Manassas, VA, USA). RPMI-1640 medium with 10% fetal bovine serum (FBS; Hyclone, South Logan, UT, USA). 100 IU/mL penicillin with 100 µg/mL streptomycin (Invitrogen, Carlsbad, CA, USA) were applied for cell culture, under 37 °C, with 5 % CO_2_.

### Lentiviral transfection

pLVX lentiviral vector was purchased from Clontech (USA). Polymerase chain reaction (PCR) was conducted using templates of IGHG1 specific shRNA and full length of IGHG1 cDNA sequences. PCR products were firstly purified using 1 % agarose gel and double digested by BamHI and EcoRI along with empty pLVX vectors. Ligation reaction was performed overnight between vector and the purified PCR products by T4DNA ligase. Ligation product was further used for the E.coli DH5α competent cells transformation. Cell clones were subsequently placed into the ampicillinum containing-LB plate and were incubated overnight at 37 °C. Positive clones were gathered and the plasmid was extracted and sequenced (Shanghai Invitrogen Biotech Co., Ltd). Afterwards, the lentiviral vectors carrying the IGHG1 shRNA / cDNA and control vectors were packaged and subsequently added into gastric cancer tumor cell groups with multiplicity of infection (MOI) of 20 (control group with empty vectors, overexpression group with IGHG1 cDNA vectors, suppressed group with IGHG1 shRNA vectors), and the vectors titer was set as 1 × 10^9^/mL. Then the fluorescent protein expression level was detected and transfection efficiency was evaluated 24–48 h post-transfection.

### Western blot

2 × 10^6^ cells per cell group were washed twice with cold PBS, re-suspended and treated by ice-cold cell lysis buffer RIPA agent (Beyotime, Shanghai, China) to extract total protein from the samples and BCA protein assay kit was used for quantification. Firstly, protein samples were separated by SDS-PAGE and 10 % separating gels and then samples were electroblotted onto PVDF membranes (Immobilon-P; Millipore, Billerica, MA). After blocking in Tris buffer (50 mM Tris, pH 7.5) containing 5 % skim milk, the membranes were incubated overnight at 4 °C with primary antibodies. Secondly, after rinsing with the Tris-Buffered Salin and Tween buffer solution (TBST; Sigma-Aldrich, St. Louis, MO, USA), they were then incubated with the secondary antibody for 2 h. Chemiluminescence was used to expose the protein bands on the membrane.

### Cellular proliferation assay

For CCK8 assay, each group of cells were seeded with density (3000 cells/well) into 96-well plates. After vector transfection, the cells were incubated for 72 h. 10 µL CCK8 was added to each well and incubated for 4 h at 37 °C. Microplate reader scanning was conducted at 450 nm to quantify cellular proliferative status. Cell survival rates were measured at three time points of the cell growth curve through the log phase of growth for each cell line.

For Edu assay, experiment was conducted according to the protocol of BeyoClick™ EdU Cell Proliferation Kit (Beyotime, Shanghai, China). Diluted EdU medium was added into samples and incubated for 2 h, and then washed in PBS and subjected to DAPI staining. All groups of samples were then inspected by inverted microscope (Olympus, Tokyo, Japan).

For spherical formation assay, 6-well plates were firstly covered using 2 mL bottom agar mixture with DMEM, 10 % fetal calf serum, and 0.6 % agar. After solidification of the bottom layer, top agar-medium mixture with 2 × 10^4^ cells from each experimental group was added and incubated at 37 °C for 2 weeks, followed by crystal violet staining. The sphere (diameter ≥ 100 μm) numbers were calculated from 5 fields per well.

### Cellular migration assay

For transwell study, each group of cell samples were firstly placed with density of 6 × 10^4^ in the upper chamber (8-µm) (Corning, Lowell, MA, USA). And the bottom chamber was added with complete medium. Then after incubation for 48 h under 37 °C, 5 % CO_2_ condition, cells in the lower chamber were fixed and stained in crystal violet solution for observation using microscope (Olympus). For the number of penetrating cells calculation, ten randomly chose fields per sample were selected.

For wound healing assay, each group of cells were respectively seeded into 6-well plate. Cellular monolayers were scraped with a sterile micropipette tip. The wounded monolayers were then washed with phosphate buffer solution (PBS) to remove debris. The distance between the two edges of the wound was calculated at three different positions. The distance between the two edges should be measured again after 48 h of incubation.

### Cellular survivability assay

Each of the cell group was respectively challenged with different concentrations of fluorouracil, cisplatin, doxorubicin for 4 h, and then cells were further washed by PBS and treated by 75 % ethanol at − 20 °C overnight. Then at room temperature, these cells were treated with Annexin V-FITC (Thermo Fisher Scientific) and propidium iodide (PI) for 20 min. Afterwards, flow cytometric analysis was performed (FACScan™, BD Biosciences, Franklin Lakes, NJ, USA) to detect apoptotic cells.

### Statistical analysis

Statistical analysis was conducted by software package (SPSS 21.0 for Windows; IBM-SPSS Inc., Chicago, IL). Data were presented as the mean SD of three independent experiments. Statistical test of differences between numerical data was performed by standard t-test. Pearson test was conducted to compare gene correlation. p < 0.05 was considered to indicate statistical significance.

## Results

### IGHG1 expression level was significantly elevated in gastric cancer samples

**Fig. 1:**
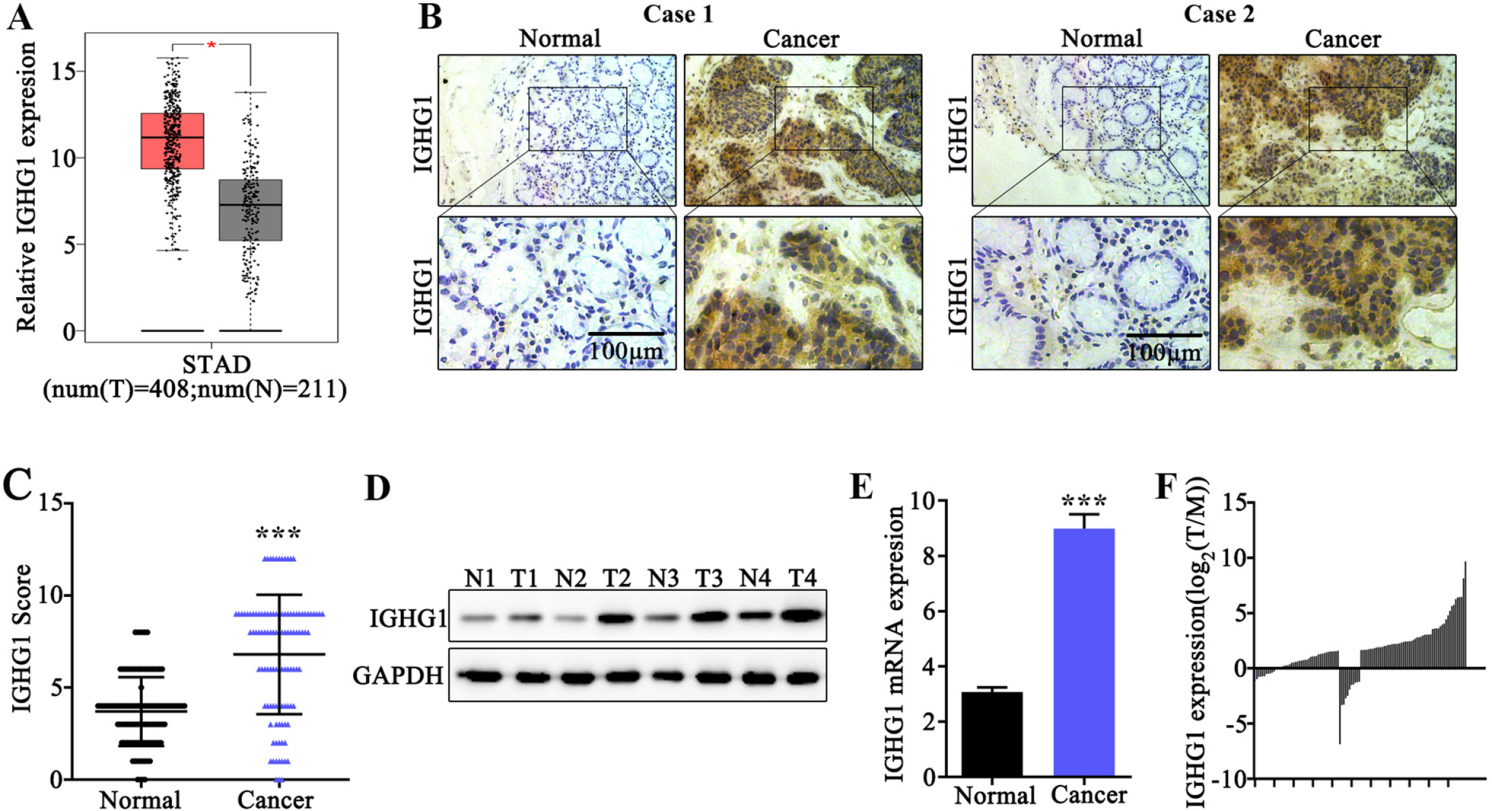
IGHG1 expression level was significantly elevated in gastric cancer samples. **A** Quantification and comparison of IGHG1 mRNA expression level in gastric cancer tumor samples and matched adjacent normal tissues utilizing data from public bioinformatic database (GEPIA); **B** Immunohistochemical (IHC) imaging of IGHG1 expression differences between clinical samples of gastric cancer tissue and normal gastric tissues from two typical clinical cases; **C** IHC imaging scores of IGHG1 expression between sample groups of gastric cancer and normal control gastric tissues; **D** Western Blot analysis of IGHG1 protein expression level in 4 pairs of clinical gastric cancer samples and matched adjacent normal tissues; **E**, **F** qRT-PCR analysis of IGHG1 mRNA expression level in clinical samples of gastric cancer and normal control tissues

**Fig. 2:**
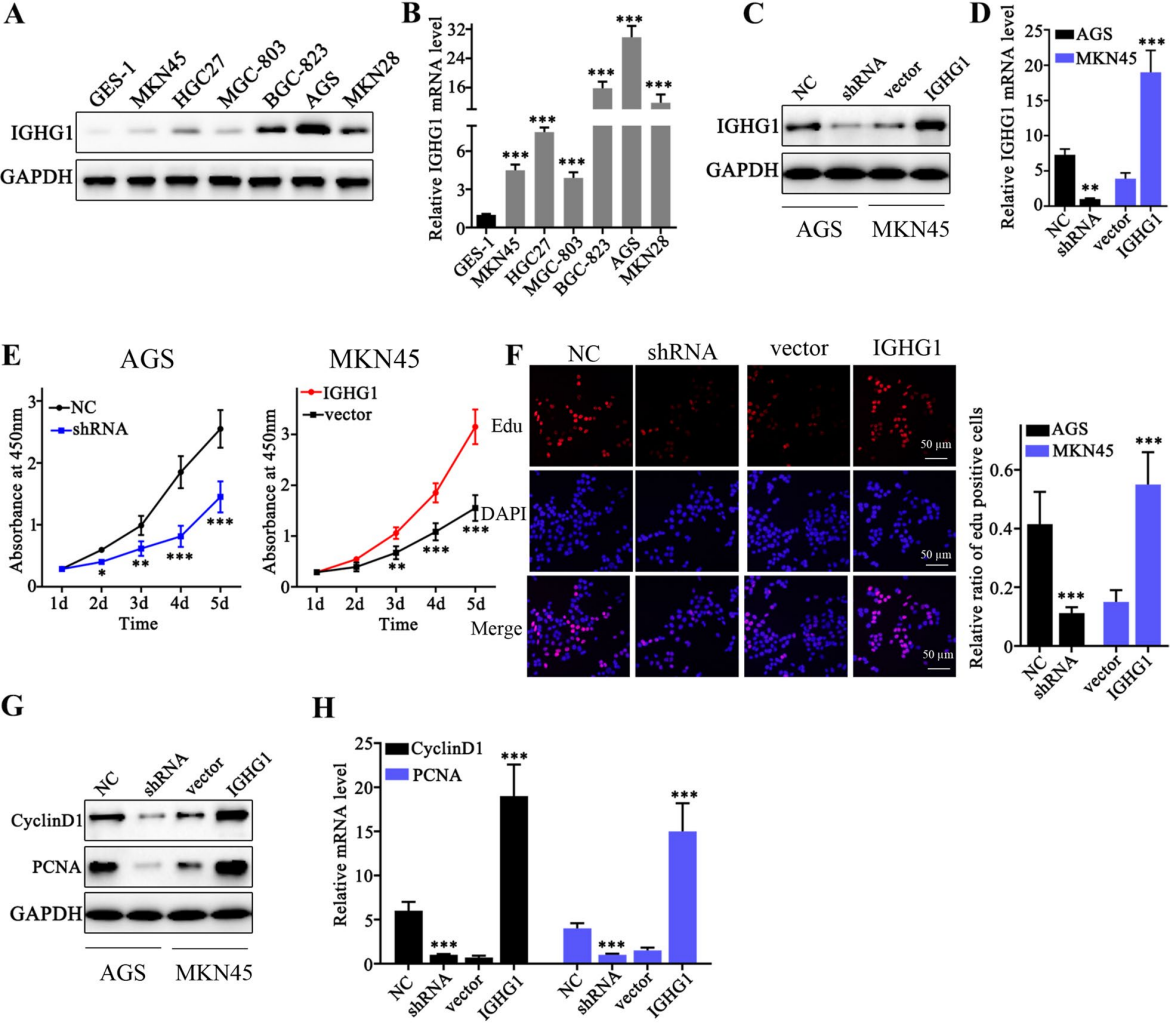
IGHG1 modulation influenced tumor cell proliferative capabilities of gastric cancer cells. **A** Western Blot analysis on IGHG1 protein expression level differences in gastric cancer cell lines (MKN45, HGC27, MGC-803, BGC-823, AGS, MKN28) and normal gastric epithelial cell line GES-1; **B** qRT-PCR detection of IGHG1 mRNA expression in gastric cancer cell lines (MKN45, HGC27, MGC-803, BGC-823, AGS, MKN28) and normal gastric epithelial cell line GES-1; **C** Design and transfection of IGHG1 specific shRNA and IGHG1 overexpression vector into AGS1 and MKN45 cell line respectively; **D** qRT-PCR analysis on the modulative effects of IGHG1 specific shRNA and overexpression vector on IGHG1 mRNA expression in AGS and MKN45 cell line; **E** CCK8 assay on AGS1 and MKN45 cellular proliferative capabilities, each cell group was respectively transfected with IGHG1 specific shRNA or IGHG1 overexpression vectors; **F** Edu assay on AGS1 and MKN45 cellular proliferative capabilities, each cell group was respectively transfected with IGHG1 specific shRNA or IGHG1 overexpression vectors;** G**,** H**. Western Blot and qRT-PCR analysis on cyclinD1 and PCNA protein and mRNA expression level in cell groups respectively transfected by IGHG1 specific shRNA and overexpression vectors

In this study, we firstly discovered IGHG1 expression pattern utilizing public bioinformatic analytic platform (GEPIA). As a result, GEP profiling data of gastric cancer tissue exhibited significantly elevated IGHG1 mRNA level compared with normal tissues (p < 0.05) (Fig. 1A). Then, to validate the above findings, immunohistochemical assay was conducted (Fig. 1B, C) on tumor and adjacent normal tissue samples from two typical gastric cancer patients. Results also demonstrated that IGHG1 protein was significantly elevated in tumor samples (p < 0.001). Moreover, subsequent western blot assay (Fig. 1D) on four pairs of gastric cancer and normal tissue samples as well as qRT-PCR study (Fig. 1E, F) on clinical gastric cancer samples in our clinical center also suggested that IGHG1 protein and mRNA level were significantly elevated in cancer tissues (p < 0.001). We then detected IGHG1 protein expression in several gastric cancer and normal epithelial cell lines. Results suggested that IGHG1 was significantly elevated in all tumor cell lines (MKN45, HGC27, MGC-803, BGC-823, AGS, MKN28), in comparison with normal gastric epithelial cell line GES-1 (Fig. 2A, B).

### IGHG1 modulation influenced tumor cell proliferative capabilities of gastric cancer cells

In order to explore the role of IGHG1 on gastric cancer cells. IGHG1 specific shRNA and overexpression vectors were constructed. qRT-PCR experiment suggested that transfection of shRNA and overexpression vector in AGS and MKN45 cell lines exhibited notably modulation on IGHG1 mRNA expression level (Fig. 2C, D). To understand the influences of IGHG1 expression modulation on the proliferation of gastric cancer cells, CCK8 and Edu assays were carried out and results both confirmed that IGHG1 expression was positively correlated with enhanced proliferation of gastric cancer cells (Fig. 2E, F). Further analysis on several genes related with cellular proliferation, including CyclinD1, and PCNA demonstrated that IGHG1 overexpression significantly elevated the expression of PCNA and CyclinD1, and vice versa (Fig. 2G, H).

### Role of IGHG1 in gastric cancer cell migration and invasion

**Fig. 3:**
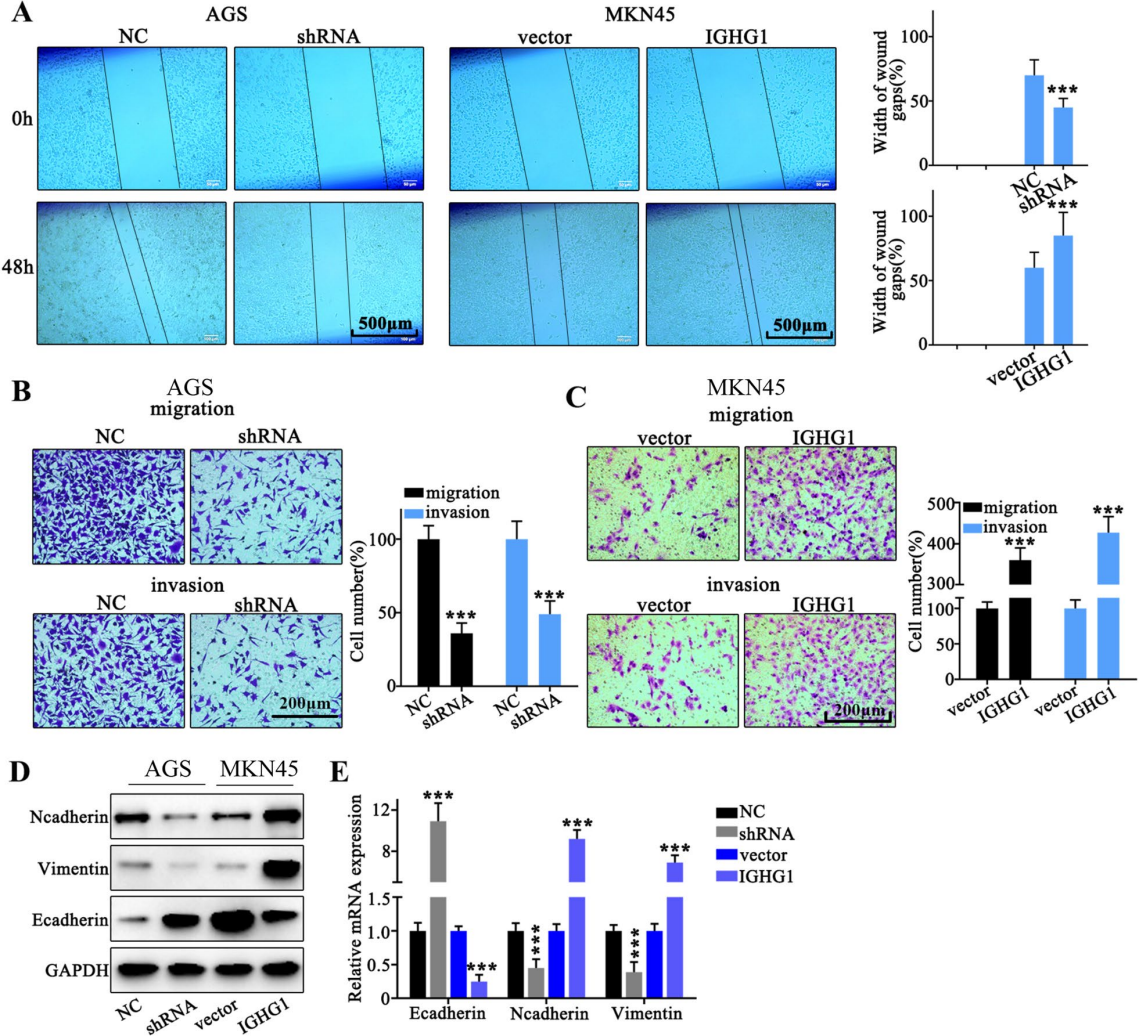
Role of IGHG1 in gastric cancer cell migration and invasion. **A** Wound healing assay on AGS and MKN45 cell line respectively transfected with IGHG1 specific shRNA and overexpression vectors; **B**, **C** Transwell assay on AGS and MKN45 cell line groups respectively transfected with IGHG1 specific shRNA and overexpression vectors; **D**, **E** Evaluation on the EMT-related protein (N-cadherin, Vimentin and E-cadherin) and mRNA expression on different cell groups respectively transfected with IGHG1 specific shRNA and overexpression vectors

To further investigate the role of IGHG1 in tumor cell migrative capabilities of gastric cancer patients, we performed wound healing assay on MKN45 and AGS cancer cell groups respectively transfected with IGHG1 overexpression and IGHG specific shRNA vectors. As a result, tumor cells with IGHG1 overexpression vector transfection exhibited significantly enhanced wound gap healing capacity compared with control group, and vice versa (Fig. 3A). Moreover, transwell assay also indicated that tumor cells with IGHG1 overexpression demonstrated that significantly increased cellular migrative and invasive capabilities compared with control groups (Fig. 3B). And IGHG1 silencing by shRNA transfection drastically suppressed tumor cell migration and invasion (Fig. 3C). To further unveil potential modulative impact of IGHG1 on tumor cell EMT (epithelial-mesenchymal transition), we utilized western blot and qRT-PCR method to detect several EMT markers (N-cadherin, Vimentin, E-cadherin). Results indicated that IGHG1 overexpression significantly increased gene involved in mesenchymal invasive phenotype (vimentin and N-Cadherin), while considerably suppressed genes involved in epithelial phenotype (E-cadherin) (Fig. 3D, E).

### Impact of IGHG1 modulation on gastric cancer cell survivability under the pressure of chemo-agents

**Fig. 4:**
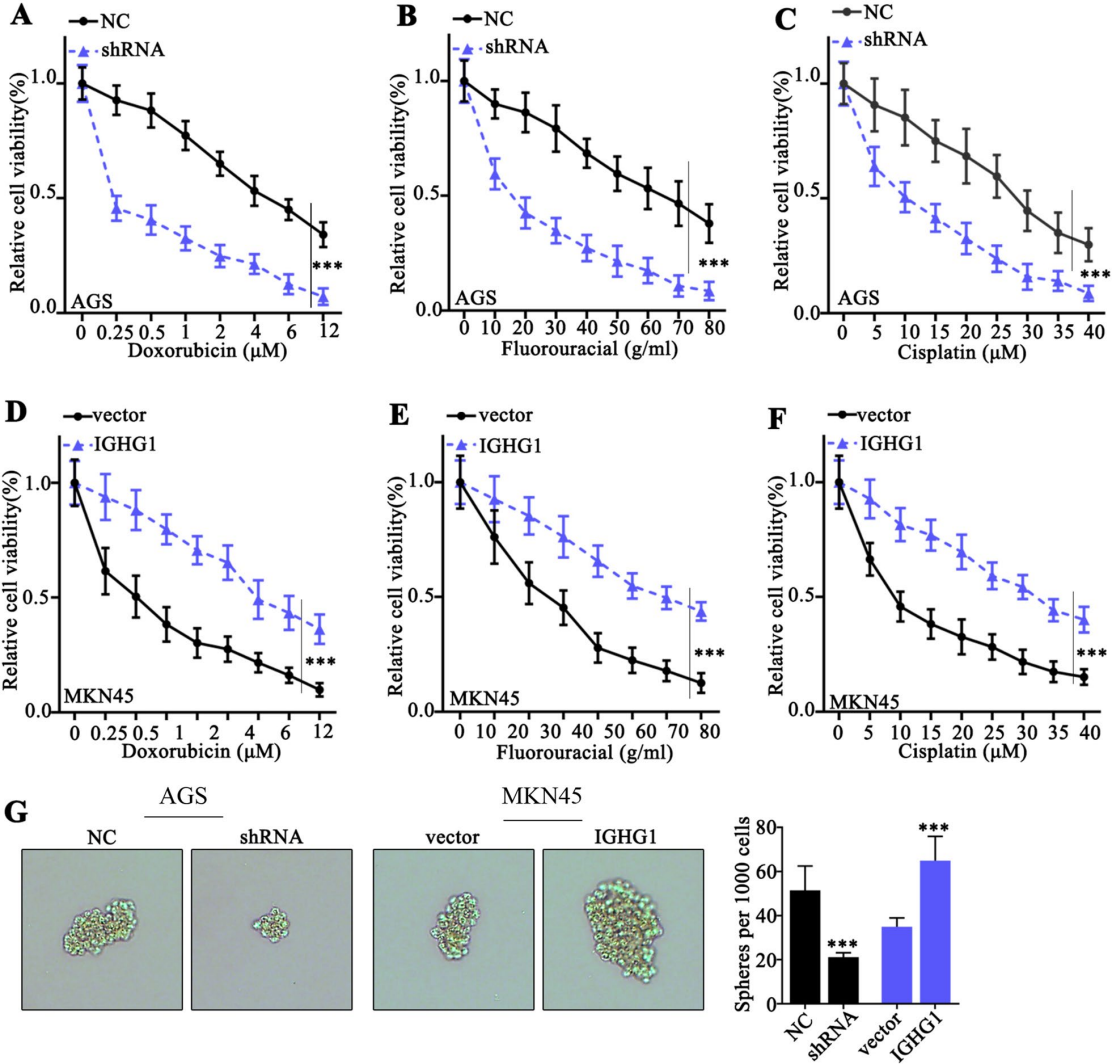
Impact of IGHG1 modulation on gastric cancer cell survivability under the pressure of chemo-agents. **A**–**F** Cellular viability assay on AGS and MKN45 cell groups respectively transfected with IGHG1 specific shRNA and overexpression vectors. Each cell group was challenged by escalated dosage of chemo-agents including doxorubicin, fluorouracil and cisplatin; **G** Sphere formation assay on AGS and MKN45 cell groups respectively transfected with IGHG1 specific shRNA and overexpression vectors

Subsequently, we performed cellular survivability assay on AGS and MKN45 cell line groups respectively transfected with IGHG1 specific shRNA and overexpression vectors. Each cell group was challenged by one of the three chemo-agents (doxorubicin, fluorouracil and cisplatin) with different concentrations. As a result, IGHG1 overexpression significantly increased cellular survivability under pressures of three chemo-agents, while IGHG1 silencing obviously inhibited cancer cell viabilities (Fig. 4A–F). Moreover, sphere formation assay provided consistent evidences that IGHG1 overexpression considerably increased tumor cell sphere formation compared with control group, and vice versa (Fig. 4G).

### β-Catenin pathway was down-stream target of IGHG1

**Fig. 5:**
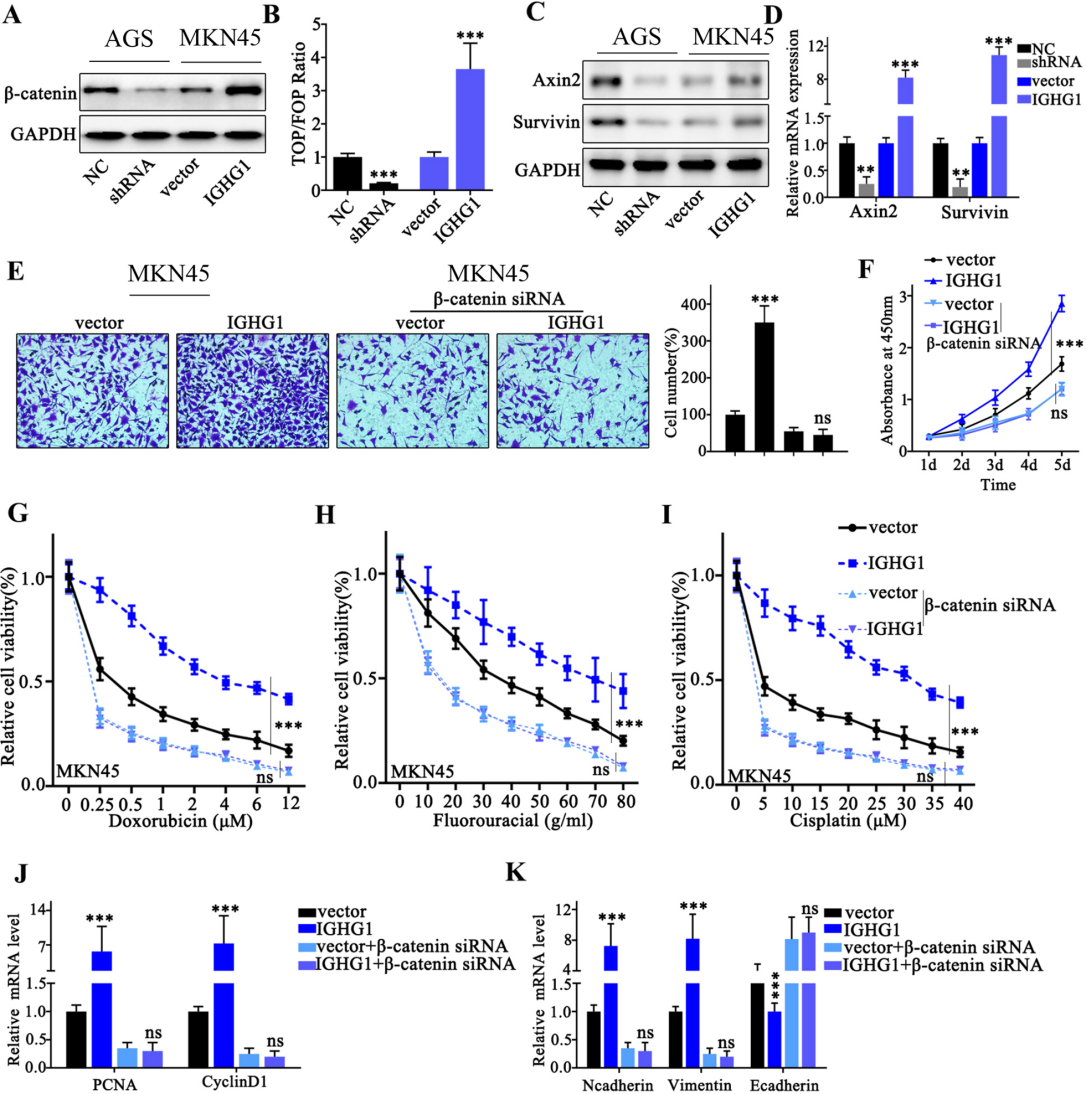
β-Catenin pathway was down-stream target of IGHG1. **A** Western Blot detection of β-Catenin protein expression on AGS and MKN45 cell groups respectively transfected with IGHG1 specific shRNA and overexpression vectors; **B** TOPflash/FOPflash detection of β-Catenin transcriptive activity of AGS and MKN45 cell groups respectively transfected with IGHG1 specific shRNA and overexpression vectors; **C**, **D** Western Blot and qRT-PCR detection on the expression levels of β-Catenin target genes (Axin 2 and Survivin); **E** Transwell assay to evaluate the MKN45 cell migration, each cell group was treated by IGHG1 overexpression vector, with or without co-transfection of β-Catenin specific siRNAs; **F** CCK8 assay to evaluate the proliferation of MKN45 cell groups treated by IGHG1 overexpression vector, with or without co-transfection of β-Catenin specific siRNAs; **G**–**I** Chemo-resistance evaluation on MKN45 cell groups treated by IGHG1 overexpression vector, with or without co-transfection of β-Catenin specific siRNAs. Cells were respectively treated by escalated dosage of doxorubicin, fluorouracil and cisplatin; **J**, **K** qRT-PCR detection of PCNA, CyclinD1, Ncadherin, Vimentin and E-Cadherin mRNA expression level in MKN45 cell groups treated by IGHG1 overexpression vector, with or without co-transfection of β-Catenin specific siRNAs

To further unveil the downstream gene regulatory network modulated by IGHG1, we detected β-Catenin protein level in cell groups respectively transfected with IGHG1 shRNA or overexpression vector. Results indicated that IGHG1 overexpression significantly elevated the protein level of β-Catenin (Fig. 5A). Then TOPflash/FOPflash assay was conducted and results indicated that IGHG1 overexpression significantly increased fluorescent activity ratio of TOPflash versus FOPflash vectors, which suggested considerably enhanced β-Catenin activity (Fig. 5B). Moreover, western blot and qRT-PCR analysis indicated that IGHG1 overexpression increased Axin2 and Survivin protein and mRNA level, which are downstream effectors of β-Catenin. While IGHG shRNA silencing significantly suppressed Axin2 and Survivin protein and mRNA level (Fig. 5C, D). Subsequently, in order to we performed transwell and cellular proliferation and chemo-resistance assay on tumor cell groups transfected IGHG overexpression vector, with or without combination of β-Catenin siRNA. As a result, IGHG1 overexpression vector transfection drastically increased tumor cell migration and proliferation compared with control group, while combinatory transfection with β-Catenin siRNA abolished tumor cell migrative and proliferative superiority (Fig. 5E, F). Moreover, chemo-resistance assay on MKN45 cell groups indicated that tumor cells transfected with IGHG1 overexpression vector demonstrated enhanced chemo-resistance compared with control cell group, under the challenges of three chemo-agents. However, combinatory transfection with β-Catenin siRNA abrogated their survival superiority exerted by IGHG1 (Fig. 5G, I). Further qRT-PCR experiments on proliferation and EMT-related genes indicated that IGHG1 overexpression noticeably enhanced PCNA, CyclinD1, as well as Vimentin mRNA expression level while significantly suppressed E-Cadherin expression level. And such effects were abolished when combinatory transfection of β-Catenin siRNA was applied (Fig. 5J, K).

### IGHG1 activates β-Catenin through inhibition of its phosphorylation process via enhancement of AKT-GSK-3β phosphorylation

**Fig. 6:**
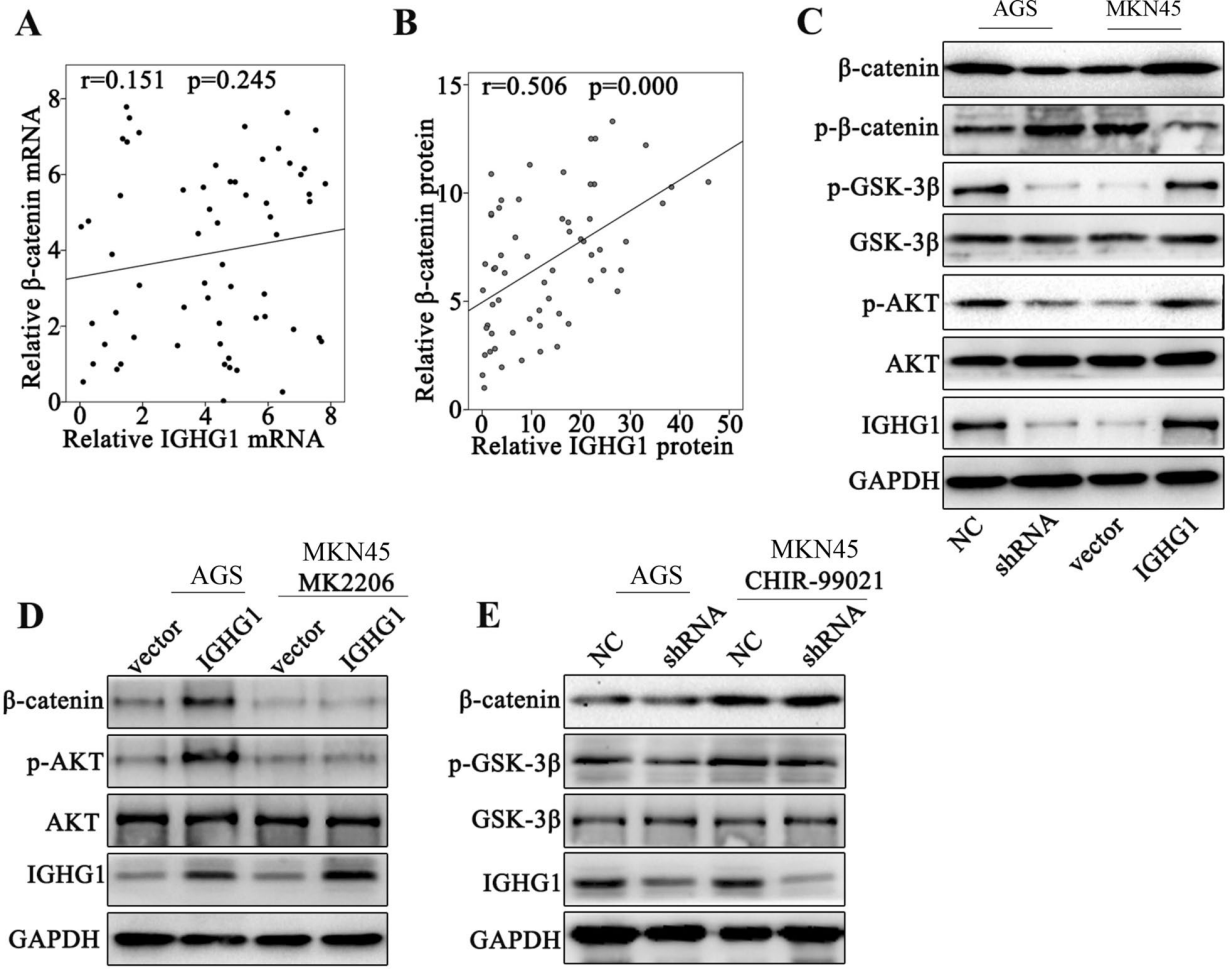
IGHG1 activates β-Catenin through inhibition of its phosphorylation process via enhancement of AKT-GSK-3β phosphorylation. **A**, **B** qRT-PCR and Western Blot analysis on the association of IGHG1 expression with β-Catenin in clinical samples of gastric cancer patients; **C** Western Blot study on protein expression level of β-Catenin, p-β-Catenin, p-GSK-3β, GSK-3β, AKT, p-AKT and IGHG1 in cell groups respectively transfected with IGHG1 shRNA and overexpression vectors; **D** Western Blot experiment on protein expression level of β-Catenin, p-AKT, AKT and IGHG1 in cell groups transfected with IGHG1 overexpression vector, with or without combination of AKT specific inhibitor, MK2206; **E** Western Blot experiment on protein expression level of β-Catenin, p-AKT, AKT and IGHG1 in cell groups transfected with IGHG1 specific shRNA, with or without combination of GSK-3β specific inhibitor CHIR-99021

In order to understand the detailed molecular mechanism of β-Catenin activation modulated by IGHG1, we further performed linear association study on β-Catenin and IGHG1 mRNA and protein levels among clinical samples from gastric cancer patients. We discovered significant association between β-Catenin and IGHG1 protein expression level but their mRNA expression levels were insignificantly correlated (Fig. 6A, B). The above phenomenon suggested that IGHG1 potentially influenced post-transcriptional modification of β-Catenin protein. Further experiments provided evidences that IGHG1 overexpression elevated β-Catenin protein level but suppressed p-β-Catenin level. Additionally, results also indicated that IGHG1 overexpression enhanced p-AKT and p-GSK-3β protein level while no significant changes were shown in AKT and GSK-3β level (Fig. 6C). The above results demonstrated that IGHG1 overexpression reduced β-Catenin phosphorylation and subsequent degradation potentially by enhancement of AKT and GSK-3β phosphorylation. To validate our theory, we utilized AKT and GSK-3β inhibitor (MK2206 and CHIR-99021) treatment in combination of IGHG1 overexpression or shRNA transfection. Our results demonstrated that MK2206 and CHIR-99021 respectively suppressed p-AKT and p-GSK-3β level and abrogated the modulative effects of IGHG1 overexpression/shRNA transfection on β-Catenin protein levels (Fig. 6D, E).

## Discussion

In this study, we demonstrated that IGHG1 upregulation was characteristic in gastric cancer cells. And IGHG1 acted as important regulator in gastric cancer cell proliferation, metastasis and chemo-resistance. Previous studies have indicated that IGHG1 demonstrated promotive effects on tumor expansion in murine pancreatic cancer models [13]. Moreover, several studies on ovarian cancer, prostate cancer also indicated that IGHG1 promoted tumor migration and metastasis via modulating EMT [14] or MEK/ERK/c-Myc pathway [15]. Our study provided consistent results that IGHG1 significantly promoted proliferative and migrative capabilities of gastric cancer cells, which could be potential therapeutic target in future treatment development. In addition, it was reported that IGHG1 could induce EMT in gastric cancer cells by regulating TGF-β/SMAD3 signaling pathway [16], which is in line with our study. Meanwhile, inhibition of IGHG1 restrained the tumor growth in nude mice and inactivated MEK/ERK/c-Myc pathway [15]. Suppression of IGHG1 leaded to growth inhibition and apoptosis induction in human prostate cancer cell [17]. Our findings enlarge the understanding of IGHG1 in tumor regulation.

In addition, in this study we reported that IGHG1 promoted gastric cancer tumor cell proliferation, migration and chemo-resistance via up-regulation of β-Catenin level. As is well known, β-Catenin plays a crucial role in Wnt/β-Catenin canonical pathway which regulates several important physiological processes including cell fate, motility, and organ formation [18]. It has been reported that β-Catenin pathway, along with nuclear factor NF-κB pathway was dysregulated in nearly 70 % of gastric cancer patients [19]. As H. pylori infection has already well been identified as important risk factor in gastric cancer pathogenesis, previous studies have indicated that several virulent factors of H. pylori were able to activate β-Catenin in cancer cells through independent phosphorylation or PI3K/Akt pathway activation [20, 21]. Our study provided novel clues in β-Catenin activation triggered by IGHG1 in gastric cancer pathogenesis, which emphasized the potential therapeutic value in targeting β-Catenin in gastric cancer treatment.

Moreover, it has been well known that GSK-3β, which is component in APC complex, could phosphorylate β-Catenin which in turn caused β-Catenin degradation. Meanwhile, activation of PI3K/Akt pathway could phosphorylate GSK3β to reduce β-Catenin loss and subsequently enhance β-Catenin activity. The results from our study demonstrated that IGHG1 upregulation in gastric cancer significantly elevated Akt and GSK-3β phosphorylation, which in turn increased β-Catenin level by suppression its degradation caused by phosphorylation. Notably, the exact molecular mechanism of IGHG1’s impact on Akt and GSK-3β phosphorylation requires future exploration. Current translational researches on therapeutic interventions by targeting Wnt/APC/β-Catenin pathway have been flourishing [22–24]. Previous reports also demonstrated effective tumor suppression through β-Catenin inhibition via adenovirus-based or antisense-based approaches [25]. However, it is still far from clinical application, future explorations are still required to discover future targeted therapy for gastric cancer patients.

It is still worth mentioning that there were indeed several limitations of our study. Firstly, the result of clinical observation in this study was based on data from single-centered clinical samples. More expanded clinical cohorts from multi-centered trials are required to validate our findings. Additionally, in this study we demonstrated the oncogenic role of IGHG1 mainly by in vitro experiments. Future researches based on in vivo animal models are needed to further confirm our findings.

## Conclusions

In this study, we demonstrated for the first time that IGHG1 upregulation was important feature of gastric cancer patients. IGHG1 promoted gastric cancer cell proliferation, invasion and chemo-resistance through up-regulation of β-Catenin level mediated by Akt/GSK-3β phosphorylation. Our study provided novel clues for the role of IGHG1 in gastric cancer oncogenesis, and enlightened future exploration for targeted therapy development.

## Supplementary Information

**Additional file 1: Table S1.** Primer Sequence.

## Data Availability

The datasets used and analyzed in the current study are available from the corresponding author in response to reasonable requests.

## Abbreviations

IGs: Immunoglobins
GSK3β: Glycogen synthase kinase 3 beta
APC: Adenomatous polyposis coli
TCF: Axin and T cell factor
LEF: Lymphoid enhancement factors
GES-1: Gastric epithelial cell
PCR: Polymerase chain reaction
PBS: Phosphate buffer solution
