# Supplementary material for: IGHG1 upregulation promoted gastric cancer malignancy via AKT/GSK-3β/β-Catenin pathway
Source: Cancer Cell Int. 2021 Jul 27;21:397. doi: 10.1186/s12935-021-02098-1 (PMC8314571; doi:10.1186/s12935-021-02098-1)
Supplement: Supplementary file 1 — Additional file 1: Table S1. Primer Sequence. [file 12935_2021_2098_MOESM1_ESM.docx]

**Supplementary Table 1**

**Primer Sequence (5' -> 3')**

IGHG1

Forward Primer GTTTTCGTCGTTGCCCTTTTAAG

Reverse Primer ACCCACTGAATGAGAATCCAGAG

GAPDH

Forward Primer GGAGCGAGATCCCTCCAAAAT

Reverse Primer GGCTGTTGTCATACTTCTCATGG

PCNA

Forward Primer CCTGCTGGGATATTAGCTCCA

Reverse Primer CAGCGGTAGGTGTCGAAGC

Ki67

Forward Primer ACGCCTGGTTACTATCAAAAGG

Reverse Primer CAGACCCATTTACTTGTGTTGGA

Ecadherin

Forward Primer CGAGAGCTACACGTTCACGG

Reverse Primer GGGTGTCGAGGGAAAAATAGG

Ncadherin

Forward Primer TCAGGCGTCTGTAGAGGCTT

Reverse Primer ATGCACATCCTTCGATAAGACTG

Vimentin

Forward Primer GACGCCATCAACACCGAGTT

Reverse Primer CTTTGTCGTTGGTTAGCTGGT
